# Investigation of Genotype by Environment Interactions for Seed Zinc and Iron Concentration and Iron Bioavailability in Common Bean

**DOI:** 10.3389/fpls.2021.670965

**Published:** 2021-05-10

**Authors:** Dennis N. Katuuramu, Jason A. Wiesinger, Gabriel B. Luyima, Stanley T. Nkalubo, Raymond P. Glahn, Karen A. Cichy

**Affiliations:** ^1^Department of Plant, Soil and Microbial Sciences, Michigan State University, East Lansing, MI, United States; ^2^USDA – ARS, U.S. Vegetable Laboratory, Charleston, SC, United States; ^3^USDA – ARS, Robert W. Holley Center for Agriculture and Health, Ithaca, NY, United States; ^4^Legumes Research Program, National Crops Resources Research Institute, Kampala, Uganda; ^5^USDA – ARS, Sugarbeet and Bean Research Unit, East Lansing, MI, United States

**Keywords:** iron bioavailability, genotype by environment interaction, biofortification, on-farm trial, *Phaseolus vulgaris* (common bean)

## Abstract

Iron and zinc malnutrition are global public health concerns afflicting mostly infants, children, and women in low- and middle-income countries with widespread consumption of plant-based diets. Common bean is a widely consumed staple crop around the world and is an excellent source of protein, fiber, and minerals including iron and zinc. The development of nutrient-dense common bean varieties that deliver more bioavailable iron and zinc with a high level of trait stability requires a measurement of the contributions from genotype, environment, and genotype by environment interactions. In this research, we investigated the magnitude of genotype by environment interaction for seed zinc and iron concentration and seed iron bioavailability (FeBIO) using a set of nine test genotypes and three farmers’ local check varieties. The research germplasm was evaluated for two field seasons across nine on-farm locations in three agro-ecological zones in Uganda. Seed zinc concentration ranged from 18.0 to 42.0 μg g^–1^ and was largely controlled by genotype, location, and the interaction between location and season [28.0, 26.2, and 14.7% of phenotypic variability explained (PVE), respectively]. Within a genotype, zinc concentration ranged on average 12 μg g^–1^ across environments. Seed iron concentration varied from 40.7 to 96.7 μg g^–1^ and was largely controlled by genotype, location, and the interaction between genotype, location, and season (25.7, 17.4, and 13.7% of PVE, respectively). Within a genotype, iron concentration ranged on average 28 μg g^–1^ across environments. Seed FeBIO ranged from 8 to 116% of Merlin navy control and was largely controlled by genotype (68.3% of PVE). The red mottled genotypes (Rozi Koko and Chijar) accumulated the most seed zinc and iron concentration, while the yellow (Ervilha and Cebo Cela) and white (Blanco Fanesquero) genotypes had the highest seed FeBIO and performed better than the three farmers’ local check genotypes (NABE-4, NABE-15, and Masindi yellow). The genotypes with superior and stable trait performance, especially the Manteca seed class which combine high iron and zinc concentrations with high FeBIO, would serve as valuable parental materials for crop improvement breeding programs aimed at enhancing the nutritional value of the common bean.

## Introduction

Iron (Fe) and zinc (Zn) malnutrition afflicts millions of people around the world. Iron and Zn are the essential trace minerals deficient in diets of people living in low- and middle-income countries with heavy consumption of plant-based staple diets (Tulchinsky, 2010). Adequate Zn and Fe nutrition is also a concern for people reliant on vegetarian diets in the developed countries. Zinc deficiency in humans can result in an impaired immune system, increased susceptibility to infections, stunting, and diminished learning ability (Krebs et al., 2014). Consumption of foods with low amounts of Fe can result in iron deficiency anemia (IDA). Majority of pregnant women and young children often suffer from IDA, which can lead to increased cases of maternal and child mortality (WHO, 2008). Dietary Fe deficiency can also result in mental impairment (infants and children), high morbidity rates, and physical incapacitation (Balarajan et al., 2011). The majority of vulnerable segments of the population for Zn and Fe deficiencies are present in low-income countries and include women, pregnant and nursing women, and infants and pre-school children (Ruel-Bergeron et al., 2015). The global burden of Zn and Fe malnutrition can result in socio-economic losses and reduced work performance and productivity, ultimately affecting national economies (Haas and Brownlie, 2021; Black, 2014).

Common bean (*Phaseolus vulgaris* L.) is a widely grown and consumed legume crop by millions of people around the world especially in Latin America and Sub-Saharan Africa, including Uganda (Akibode and Maredia, 2011; Larochelle et al., 2017). Common bean belongs to two gene pools (Middle American and Andean) that occurred during two separate domestication events in Central and South America (Bitocchi et al., 2013). The two gene pools can be distinguished using morphological, physiological, and molecular characteristics (Kami and Gepts, 1994; Tohme et al., 1996; Bitocchi et al., 2013).

Common bean is a major source of protein, micronutrients (Zn and Fe), dietary fiber, and vitamins (Hayat et al., 2014). Seed Fe and Zn concentration has been reported to vary by common bean gene pool where Andean genotypes accumulate more Fe than the Middle American germplasm. Conversely, Middle American genotypes tend to accumulate more seed Zn than the Andean common bean accessions (Blair et al., 2010a). Common bean has a wide native variability for Zn and Fe concentration, where seed Zn ranging from 20 to 59 μg g^–1^ and seed Fe values of 34 to 99 μg g^–1^ in both raw and cooked samples have been reported (Beebe et al., 2000; Islam et al., 2002; Katuuramu et al., 2018; Caproni et al., 2020). The presence of wide variability for seed Zn and Fe along with high levels of consumption has made the crop a strong target for biofortification programs in Eastern Africa (Nestel et al., 2006).

Several countries in Eastern Africa, particularly Uganda, Democratic Republic of Congo, Rwanda, Burundi, and Tanzania, have been participating in HarvestPlus biofortification programs focused on the production and consumption of nutrient-enhanced staple crops including high-mineral beans for the past decade (Bouis and Saltzman, 2017; Bouis, 2018). Common bean nutrition breeding goals have mainly focused on development and release of genotypes with high levels of Fe and Zn (Saltzman et al., 2017). High levels of Fe, according to HarvestPlus, have been defined as values greater than the assumed average of 50 μg g^–1^, with 90–94 μg g^–1^ being the target value. For Fe- and Zn-biofortified crops to be effective in alleviating deficiency, the minerals must be available for absorption and complexed and thus non-exchangeable with the uptake transporters in the human intestine (Petry et al., 2010). Iron bioavailability (FeBIO) or the ability for Fe present in food to be released following ingestion and digestion for absorption and biological functioning of the human body should be an integral process of the common bean biofortification breeding process (Hurrell and Egli, 2010). To date, the methodology for Zn bioavailability is limited to *in vivo* studies (Dias et al., 2018) as *in vitro* studies have not been effective at predicting Zn uptake (Cheng et al., 2012).

The majority of the Ugandan population, especially those living in rural areas, often rely on the repeated consumption of diets composed of staple food crops including common bean (Larochelle et al., 2015; UBOS, 2017). These rural population also suffer the burden of Zn and Fe deficiencies, especially pregnant women, nursing mothers, and children under 5 years old (UBOS, 2016). Introduction of new bean varieties and identification of varieties already in the food system that deliver more bioavailable Fe and Zn represent an effective approach to address Fe and Zn deficiency in the Ugandan communities. Since farmers in the rural areas are the direct producers and end-users of common bean and depend on this crop to provide micronutrients in their diets, it is important to understand the role genotype and production environment play in seed nutrient density and bioavailability.

Plants accumulate trace minerals such as Zn and Fe as well as minerals such as calcium, potassium, phosphorus, and magnesium into the edible parts like leaves and seeds (Baxter et al., 2012). Seed nutrient composition in plants can be affected by genotype, weather, soil nutrient composition, uptake by the roots, translocation, and redistribution within the plant, followed by remobilization and mineral loading into the seeds (Kobayashi and Nishizawa, 2012; Baligar and Fageria, 2015). Evaluation of genotype by environment and phenotype performance stability for seed nutritional quality traits across different agro-ecological conditions has not been adequately examined in common bean. This study was conducted to assess the magnitude of genotype and genotype by environment interactions for seed Zn and Fe concentration and FeBIO of common bean genotypes grown at nine on-farm locations in Uganda.

## Materials and Methods

### Germplasm Materials

The study was comprised of nine genotypes and three farmers’ local check varieties (Table 1). The nine genotypes were selected based on screening and genetic study results of a larger Andean germplasm collection (over 200 genotypes) for cooking time, iron and zinc concentration, and FeBIO (Cichy et al., 2015; Katuuramu et al., 2018). The majority of the genotypes belonged to the Andean gene pool except Amarelo Cela, Chijar, and PR0737-1. Most of the genotypes were collected from sub-Saharan Africa except for Blanco Fanesquero, Chijar, and PR0737-1. One genotype had a white seed coat color, four were yellow, and four had a red mottled grain color (Table 1). Three of the farmers’ preferred local check genotypes (NABE-15, NABE-4, and Masindi yellow) were sourced in Uganda and included in the field study and mineral analysis. All genotypes and local checks were pure lines. Additional detailed information about the local check genotypes like germplasm release institutions and dates plus specific variety trait ontology can be found in Katuuramu et al. (2020).

**TABLE 1:** Description of the experimental common bean genotypes evaluated over 2 years across nine on-farm locations in Uganda.

Genotype name	Gene pool	Region of origin	Country of origin	Cultivation status	Seed type	Growth habit
Blanco Fanesquero	Andean	South America	Ecuador	Variety	White	Determinate
Ervilha	Andean	Southern Africa	Angola	Landrace	Yellow	Determinate
PI527538	Andean	East Africa	Burundi	Landrace	Yellow	Determinate
Cebo Cela	Andean	Southern Africa	Angola	Landrace	Yellow	Indeterminate
Amarelo Cela	MA	Southern Africa	Angola	Landrace	Yellow	Indeterminate
Maalasa	Andean	East Africa	Tanzania	Landrace	Red mottled	Determinate
Rozi Koko	Andean	East Africa	Tanzania	Landrace	Red mottled	Determinate
Chijar	MA	Caribbean	Puerto Rico	Landrace	Red mottled	Indeterminate
PR0737-1	Admix	Caribbean	Puerto Rico	Variety	Red mottled	Indeterminate
Local checks:						
NABE-15	Andean	East Africa	Uganda	Variety	Cream mottled	Determinate
NABE-4	Andean	East Africa	Uganda	Variety	Red mottled	Determinate
Masindi yellow	Andean	East Africa	Uganda	Landrace	Yellow	Determinate

*MA, Middle American common bean gene pool.*

### Field Experiments

The field research was conducted at nine on-farm locations in four districts (Hoima, Kamuli, Rakai, and Masaka) spanning different agro-ecological zones in Uganda (MAAIF, 2010). The nine on-farm field locations included Kakindo, Kyamalera, and Tugonzagane in Hoima district (Table 2). The Kamuli district locations were Katugezeko, Tweweyo, and Tweyunge, while the Rakai district was comprised of two locations, i.e., Agali-awamu and Kiyovu (Table 2). The Masaka district had one on-farm location of Balitwewunya (Table 2). Soil sampling was performed just before planting to assay for pH, organic matter, nitrate–nitrogen (NO_3_–N), Zn, Fe, Bray-1 extractable phosphorus (P), potassium (K), calcium (Ca), and magnesium (Mg) based on published protocols (Nathan and Gelderman, 2012). Two locations (Kakindo and Agali-awamu) had sandy clay, while four sites (Kyamalera, Tweweyo, Tweyunge, and Kiyovu) had sandy clay loam soil type. The locations Tugonzagane and Balitwewunya had clay soils, while the location Katugezeko had clay loam soil type (Table 2). The field experiments were performed as a randomized complete block design with two replications for the field seasons of 2015 and 2016. At crop maturity in each field season, the plants were harvested and shelled manually to minimize any metal contamination and processed for agronomic and cooking time data as described in Katuuramu et al. (2020).

**TABLE 2:** Description of the nine on-farm locations used for the common bean genotype by environment study in Uganda.

District name	Annual rainfall range (mm)	Annual temperature range (°C)	Agro-ecological zone	Location name	Geographic coordinates		Altitude (m asl)	Soil type
Hoima	800–1,400	15–30	Grass land savanna	Kakindo	N01°28.54′	E031°25.46′	1,228	Sandy clay
				Kyamalera	N01°29.47′	E031°26.99′	1,174	Sandy clay loam
				Tugonzagane	N01°16.93′	E031°17.77′	1,138	Clay
Kamuli	800–1,300	16–31	Tall savanna	Katugezeko	N00°50.60′	E033°12.11′	1,127	Clay loam
				Tweweyo	N00°54.79′	E033°01.33′	1,086	Sandy clay loam
				Tweyunge	N00°53.77′	E032°59.94′	1,061	Sandy clay loam
Rakai	850–2,125	15–27	Tropical rain forest	Agali-awamu	S00°34.87′	E031°34.19′	1,233	Sandy clay
				Kiyovu	S00°43.58′	E031°29.27′	1,215	Sandy clay loam
Masaka	850–2,125	15–27	Tropical rain forest	Balitwewunya	S00°25.54′	E031°38.14′	1,249	Clay

### Seed Handling and Preparation

Prior to cooking, a total of 150 seeds for each genotype from both field seasons were placed into paper envelopes and stored at 4°C and relative humidity of 75% to attain a seed moisture content of 10–12%. The moisture-equilibrated raw seeds were pre-soaked in distilled water for 12 h at room temperature before cooking time using a Mattson pin drop cooking device under a steady boil at 100°C (Wang and Daun, 2005; Katuuramu et al., 2020). The cooked seeds were cooled to room temperature and then frozen to –80°C prior to freeze-drying (VirTis Research Equip., Gardiner, NY, United States). The lyophilized cooked common bean samples were ground to a fine powder with a Kinematica Polymix^®^ analytical hammer mill (PX-MFC 90D, Bohemia, NY, United States) fitted with a 0.5-mm sieve and stored in sealed polypropylene containers at room temperature.

### Common Bean Mineral Analysis

For mineral analysis, a 500-mg sample of cooked/lyophilized/milled samples was pre-digested with 3 ml of a concentrated ultra-pure nitric acid and perchloric acid mixture (60:40 v/v) for 16 h at room temperature in boro-silicate glass tubes. All samples were pre-digested and measured with 0.5 μg ml^–1^ of yttrium (final concentration) to ensure batch-to-batch accuracy and to correct for matrix inference during digestion. After pre-digestion, the samples were placed in a digestion block (Martin Machine, Ivesdale, IL, United States) and heated incrementally over 4 h to a temperature of 120°C with refluxing. Following incubation at 120°C for 2 h, 2 ml of concentrated ultra-pure nitric acid was subsequently added to each sample before raising the digestion block temperature to 145°C for an additional 2 h. The temperature of the digestion block was then raised to 190°C and maintained for at least 10 min to evaporate any remaining liquid. The digested samples were re-suspended in 20 ml of ultrapure water prior to analysis using the inductively coupled plasma-atomic emission spectroscopy machine (Thermo iCAP 6500 Series, Thermo Scientific, Cambridge, United Kingdom). Quality control standards (High Purity Standards, North Charleston, SC, United States) were measured after every 10 samples. The internal standard yttrium was purchased from High Purity Standards (10M67-1).

### Quantification of Seed Iron Bioavailability

An established *in vitro* digestion/Caco-2 cell culture model was used to assess the FeBIO of the common bean genotypes after cooking (Glahn et al., 1998; Tako et al., 2016). A 500-mg sample of cooked/lyophilized/milled samples was subjected to a simulated gastric and intestinal digestion as described previously (Glahn et al., 1998, 2017). The bioassay was performed according to the methods described in Glahn et al. (1998). The bioassay measures FeBIO according to the following principle: in response to increases in cellular Fe concentrations, Caco-2 cells produce more ferritin protein; therefore, FeBIO was determined as the increase in Caco-2 cell ferritin production (ng ferritin per mg of total cell protein) after exposure to a digested sample (Glahn et al., 1998; Tako et al., 2016). Ferritin was measured by enzyme linked immunoassay (Human Ferritin ELISA kit S-22, Ramco Laboratories Inc., Stafford, TX, United States), and total cell protein concentrations were quantified using the Bio-Rad DC^TM^ protein assay kit (Bio-Rad Laboratories Inc., Hercules, CA, United States).

To confirm the responsiveness of the Caco-2 bioassay, each experiment was run with several quality controls. These include a blank digest, which is only physiologically balanced saline and gastrointestinal enzymes. The blank digest was used to ensure that there was no Fe contamination in the bioassay. The ferritin values of Caco-2 cells exposed to the blank digest averaged 2.53 ± 0.81 ng ferritin/mg cell protein [mean ± standard deviation (SD)] over the course of several months of experiments. The responsiveness of the bioassay was monitored by using (1) a blank digest with FeCl_3_ (66 μM) and (2) a blank digest of FeCl_3_ (66 μM) plus the addition of 1.3 mM ascorbic acid (Sigma Aldrich Co., St. Louis, MO, United States). The ferritin values for FeCl_3_ digest and FeCl_3_ digest with ascorbic acid averaged 42.9 ± 4.5 and 541 ± 27 ng ferritin/mg cell protein (mean ± SD), respectively.

The quality controls cannot be utilized as a reference standard because they do not contain similar food matrix properties as the cooked common bean samples. Therefore, a cooked/lyophilized/milled navy common bean control (commercial variety Merlin) was run with each assay as a reference standard to index the ferritin/total cell protein ratios of the Caco-2 cells over the course of several months of research. The ferritin values for the white common bean control averaged 12.5 ± 1.7 ng/mg cell protein (mean ± SD). The iron concentrations for the navy Merlin control averaged 74 ± 2.2 μg g^–1^ on a dry weight basis over the course of the Caco-2 cell experiments. Merlin navy common bean was used as a control because it is widely available across the United States and can be grown, cooked, and processed in large quantities. Merlin has also been used previously as a control in the screening of the Andean Diversity Panel for seed FeBIO (Katuuramu et al., 2018). Additionally, white common bean genotypes have been reported to exhibit higher levels of FeBIO (Tako and Glahn, 2011).

### Phenotypic Data Analysis

The analysis of variance (ANOVA) for all sources of variation in the statistical model was conducted using the PROC MIXED statement in the statistical analysis software SAS, version 9.4, (SAS Institute, 2013). Pearson correlation coefficients among traits across locations and years were determined using the PROC CORR command in SAS, version 9.4. The statistical model used for ANOVA and computing variance components in SAS is shown below:

$$Y_{i\,j\,k\,m}=\mu +G_{i}+L_{j}+S_{k}+{\text{GL}}_{i\,j}+{\text{GS}}_{i\,k}+{\text{LS}}_{j\,k}+{\text{GLS}}_{i\,j\,k}+\text{rep}\,{\left(\text{LS}\right)}_{j\,k\,m}+\varepsilon_{i\,j\,k\,m}$$

where Y_ijkm_ is the response variable like seed zinc or iron concentration of the ith common bean genotype in the mth replication of the jth location in the kth season; μ is the grand mean; G_i_, L_j_, S_k_, GL_ij_, GS_ik_, LS_jk_, and GLS_ijk_ are effects of the ith genotype, jth location, kth season, and their respective interactions; rep(LS)_jkm_ denotes the effect of the mth replication nested within the interaction term of the jth location and kth season; and ε_ijkm_ is the error term assumed to be normally distributed with mean = 0. The effects of G, L, and GL were treated as fixed, while the remaining effects were treated as random to estimate Fisher’s protected least significant difference and to compare means among the common bean genotypes and locations for each trait. The variance components for computing broad-sense heritability (H^2^) estimates on a plot basis were generated using the PROC VARCOMP procedure in SAS, version 9.4, using the restricted maximum likelihood estimation method (SAS Institute, 2013). Broad-sense heritability across environments on a plot basis was computed as described in Holland et al. (2003) and shown in the equation below:

$$H^{2}=\frac{\text{Var}\,\left(G\right)}{\text{Var}\,\left(G\right)+\mathrm{Var}\,\left(\text{GL}\right)+\text{Var}\,\left(\text{GS}\right)+\mathrm{Var}\,\left(\text{GLS}\right)+\mathrm{Var}\,\left(\text{Error}\right)}$$

where Var(*G*) is genotypic variance, Var(GL) is the genotype by location variance, Var(GS) is the genotype by season variance, Var(GLS) is the genotype by location by season variance, and Var(Error) is the residual/experimental error variance. Phenotypic variability explained (PVE) was computed as a ratio of the type III sum of squares for each source of variation in the statistical model above divided by the total sum of squares from the ANOVA table and multiplied by 100 (i.e., expressed as a percentage).

## Results

### Analysis of Variance

The most important sources of variation for seed Zn concentration were genotype (28.0% of PVE) and location (26.2% of PVE), followed by the interaction term between location and field season (14.7% of PVE; Table 3). Seed Fe concentration was largely controlled by genotype (25.7% of PVE), followed by location (17.4% of PVE). The interaction terms *L* × *S* and *G* × *L* × *S* each contributed 11.3 and 13.7% of PVE to the total variation observed in seed Fe concentration of the cooked common bean samples (Table 3). The variability in seed FeBIO was explained to a large extent by genotype (68.3% of PVE). Location, *G* × *L*, and *L* × *S* contributed 6.0, 6.9, and 7.4% of PVE, respectively (Table 3).

**TABLE 3:** ANOVA showing the mean squares and percentage of total variance explained for seed zinc and iron concentration and iron bioavailability of the cooked common bean genotypes evaluated for two field seasons at nine on-farm locations in Uganda.

Source of variation	df	Traits					
		Zn (μg g^–1^)		Fe (μg g^–1^)		FeBIO (% of Merlin navy bean control)	
		Mean square	% PVE	Mean square	% PVE	Mean square	% PVE
G	9	243.1*	28.0	1,093.8*	25.7	18,061.9*	68.3
L	8	255.9*	26.2	835.9*	17.4	1,783.9*	6.0
S	1	95.7*	1.2	3,336.9*	8.7	1,060.9*	0.4
G × L	72	9.3*	8.6	59.8*	11.2	229.0*	6.9
G × S	9	14.9*	0.02	83.6*	2.0	211.3*	0.8
L × S	8	143.0*	14.7	542.1*	11.3	2,197.3*	7.4
Rep (L × S)	18	6.9*ns*	1.6	27.5*ns*	1.3	200.4*	1.5
G × L × S	72	8.2*	7.6	72.7*	13.7	159.2*	4.8

df, degrees of freedom; % PVE, percentage of phenotypic variability explained; and ns, not significant. *P value ≤ 0.005.

### Evaluation of Genotype and Environment Performances

**FIGURE 1:**
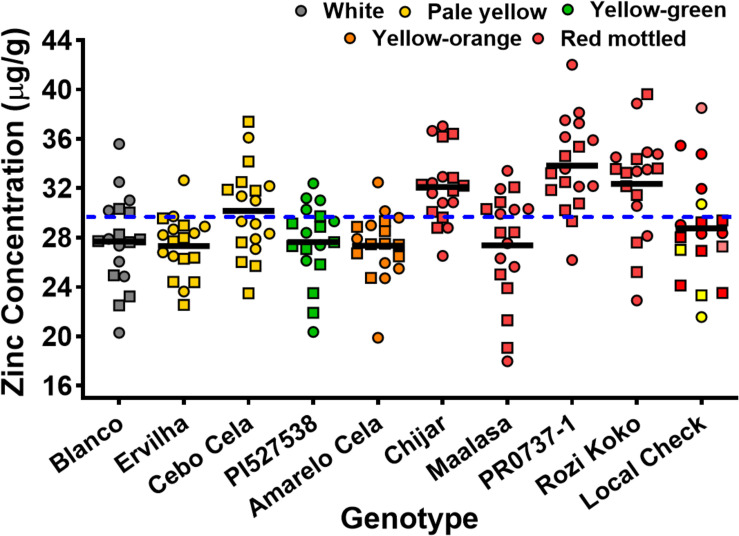
Scatter plot showing seed Zn concentrations of common bean genotypes grown at nine on-farm locations across three agro-ecological zones in Uganda. The zinc concentrations are measured as micrograms per gram of cooked, lyophilized, and milled whole seed (dry weight basis). Each value represents the mean of two field replicates per genotype from each location for field seasons 2015 (squares) and 2016 (circles). Local checks Masindi yellow and NABE-4 are distinguished from NABE-15 by their respective yellow and pink colors. The black bars indicate the mean Zn concentration for each genotype. The blue hyphenated line indicates the overall mean for all the study genotypes across the 2015 and 2016 field seasons. Blanco, Blanco Fanesquero.

**FIGURE 2:**
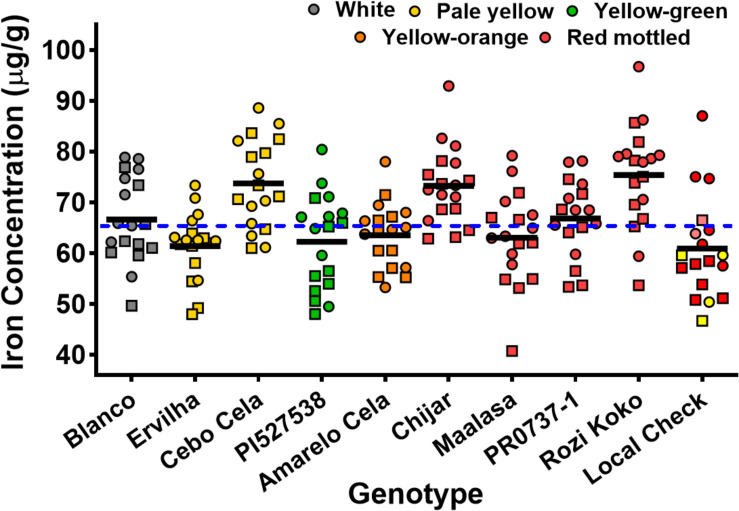
Scatter plot showing the seed Fe concentrations of common bean genotypes grown at nine on-farm locations across three agro-ecological zones in Uganda. The iron concentrations are measured as micrograms per gram of cooked, lyophilized, and milled whole seed (dry weight basis). Each value represents the mean of two field replicates per genotype from each location for field seasons 2015 (squares) and 2016 (circles). Local checks Masindi yellow and NABE-4 are distinguished from NABE-15 by their respective yellow and pink colors. The black bars indicate the mean Fe concentration for each genotype. The blue hyphenated line indicates the overall mean for all the study genotypes across the 2015 and 2016 field seasons. Blanco, Blanco Fanesquero.

**FIGURE 3:**
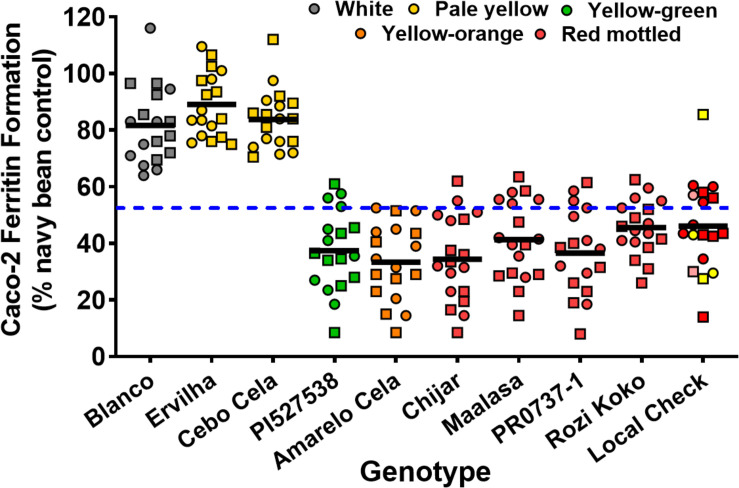
Scatter plot depicting the *in vitro* iron bioavailability of common bean genotypes grown at nine on-farm locations across three agro-ecological zones in Uganda. The iron bioavailability in cooked, lyophilized, and milled whole seed is expressed as Caco-2 cell ferritin formation (ng ferritin/mg total cell protein) relative to a Merlin white navy bean control that was run with each assay. Each value represents the mean of two field replicates per genotype from each location for field season 2015 (squares) and 2016 (circles). Local checks Masindi yellow and NABE-4 are distinguished from NABE-15 by their respective yellow and pink colors. Black bars indicate the mean iron concentration for each genotype. Blue hyphenated line indicates the overall mean for all the study genotypes across the 2015 and 2016 field seasons. Blanco, Blanco Fanesquero.

Seed Zn concentration among genotypes ranged from 18.0 to 42.0 μg g^–1^ (Figure 1 and Supplementary Table 2). The highest seed Zn concentration was observed in genotype PR0737-1, a red mottled variety with an indeterminate growth habit from the Caribbean (Figure 1 and Table 1). The genotypes Rozi Koko, Chijar, and local check variety (NABE-4) were all red mottled and had the second highest amount of seed Zn concentration (Figure 1, Table 4, and Supplementary Table 2). The seed Fe concentration among genotypes varied from 40.7 to 96.7 μg g^–1^ (Figure 2 and Supplementary Table 3). The highest seed Fe concentration was recorded among genotype Rozi Koko, a red mottled determinate landrace from Tanzania (Table 1). The genotypes Cebo Cela and Chijar had the second highest amounts of seed Fe concentration (Figure 2, Table 4, and Supplementary Table 3). Both Cebo Cela (yellow) and Chijar (red mottled) are landraces with an indeterminate growth habit (Table 1). Seed FeBIO among genotypes varied from 8 to 116% of Merlin navy common bean control (Figure 3 and Supplementary Table 4). The genotypes Blanco Fanesquero, Ervilha, and Cebo Cela had the highest amount of bioavailable Fe based on the Caco-2 cell bioassay (Table 4 and Supplementary Table 4). Blanco Fanesquero is a white kidney variety from Ecuador, while Ervilha and Cebo Cela are both yellow-colored landraces from Angola with a determinate growth habit (Table 1).

**TABLE 4:** Genotype means for the nutritional quality traits of the cooked common bean genotypes evaluated across nine on-farm field sites for 2 years in Uganda.

Genotype name	Zn (μg g^–1^)	Fe (μg g^–1^)	FeBIO (% of Merlin navy bean control)
Blanco Fanesquero	27.7	66.6	81.6
Ervilha	27.3	61.4	89.0
PI527538	27.6	62.2	37.4
Cebo Cela	29.9	73.7	83.8
Amarelo Cela	27.4	63.5	33.4
Maalasa	27.4	63.0	41.3
Rozi Koko	32.4	75.4	45.6
Chijar	32.1	73.2	34.4
PR0737-1	34.5	67.4	37.6
Local checks:			
NABE-15	29.2	62.8	49.5
NABE-4	32.9	65.2	43.5
Masindi yellow	25.5	52.9	36.9
LSD (*α* = 0.05)	1.0	2.1	3.5

*LSD, least significant difference used to compare genotype performances for the measured traits.*

Across all the study environments, the mean performance for seed Zn concentration ranged from 23.8 to 33.1 μg g^–1^, with genotypes grown at location Tugonzagane in Hoima district accumulating the most seed Zn, while genotypes harvested from location Tweyunge in Kamuli district had the lowest seed Zn (Table 5). Genotypes harvested from location Kiyovu in Rakai district accumulated the most seed Fe of 74.1 μg g^–1^ (Table 5). Genotypes grown at locations Tweweyo and Agali-awamu accumulated the least amount of seed Fe at 58.7 and 61.9 μg g^–1^, respectively (Table 5). Genotypes harvested from location Tugonzagane had the most bioavailable seed Fe, while genotypes from Katugezeko and Kiyovu had the least FeBIO (Table 5).

**TABLE 5:** Environmental means for the nutritional quality traits across the cooked common bean genotypes evaluated at nine on-farm locations for 2 years in Uganda.

Location name	Zn (μg g^–1^)	Fe (μg g^–1^)	FeBIO (% of Merlin navy bean control)
Kakindo	30.1	65.0	56.0
Kyamalera	30.7	68.2	58.9
Tugonzagane	33.1	68.4	64.3
Katugezeko	31.2	69.7	46.2
Tweweyo	23.8	58.7	47.5
Tweyunge	28.7	69.3	57.9
Agali-awamu	28.9	61.9	49.8
Kiyovu	29.0	74.1	44.5
Balitwewunya	30.0	65.5	52.1
LSD (*α* = 0.05)	1.0	2.0	3.3

*LSD, least significant difference used to compare environmental performances for the measured traits.*

### Correlation Among Traits

Pearson correlation analysis showed that seed Zn had a moderately high correlation with seed Fe concentration (*r* = 0.61; *P* = 0.0001) but not correlated with FeBIO. Seed Fe concentration was also not correlated to FeBIO (Table 6).

**TABLE 6:** Pearson correlation coefficients among traits across the nine on-farm locations and two field seasons.

Traits	Zn	Fe	FeBIO
Zn	–	0.61^*a*^	0.01 ns
Fe		–	0.09 ns
FeBIO			–

*^*a*^Significant at the 0.0001 probability level. ns, not significant.*

## Discussion

Uganda has one of the highest per capita consumption rates of common bean in sub-Saharan Africa, but the country also has one of the highest prevalence rates of IDA, affecting especially women, children, and infants (Larochelle et al., 2015; UBOS, 2016, 2017). In sub-Saharan Africa, Uganda is one of the major producing countries of common bean across the different agro-ecological zones present in the country, and majority of the seed types evaluated in this research are what is often consumed (UBOS, 2017). Consequently, production and consumption of high-Fe common beans have been promoted by HarvestPlus and local and international non-governmental organizations over the past 10 years (Petry et al., 2015; Bouis and Saltzman, 2017).

Both seed Fe and Zn concentration were largely affected by the genotype and location effects (Table 3). Soil chemical composition properties and weather patterns have been known to influence nutrient uptake in plants, as these affect nutrient solubility and availability in the root zone (Manzeke et al., 2012). Moreover, available Fe and Zn for plant uptake are mainly controlled by soil organic matter fraction, pH, aeration, and interactions with other soil nutrients (Charlson and Shoemaker, 2006). In our study, one of the sites (Kiyovu) that resulted in high seed Fe also had the highest mean soil Fe over the two field seasons. A similar pattern was observed for seed Zn where locations Tugonzagane and Katugezeko with high mean seed Zn also had high mean soil Zn concentrations at planting.

The local check bean genotypes NABE-15 and Masindi yellow accumulated low levels of seed Fe concentrations compared to most of the test genotypes. However, the red mottled local check variety (NABE-4) had high levels of Zn compared to majority of the tested genotypes (Table 4). All the local check genotypes had low levels of seed FeBIO. The local check genotypes have been reported to be fast cooking and are of preferable seed coat color (red mottled or yellow), which are among the reasons why Ugandan growers and consumers like them (Katuuramu et al., 2020). Screening germplasm for seed FeBIO has not been included in common bean biofortification breeding efforts. Genotypes have been instead assumed to be nutritious based solely on seed Fe concentration. The ability for Fe and Zn to be absorbed upon ingestion and digestion is an important aspect of human nutrition and health (Hambidge et al., 2010; Hurrell and Egli, 2010). Common bean genotypes often accumulate Fe and Zn binding compounds like phytic acid and certain classes of seed coat polyphenols that limit nutrient release following consumption (Petry et al., 2010; Hart et al., 2017, 2020).

Our results showed that genotype largely controlled the variability observed in FeBIO (68.3%), and yellow-colored (Ervilha and Cebo Cela) and white kidney (Blanco Fanesquero) had the highest values compared to the other genotypes. Moreover, broad-sense heritability across environments was 46.5, 38.7, and 79.5% for seed Zn, Fe, and FeBIO, respectively. The moderate to high heritability estimates uncovered in this research suggest the presence of strong genetic control and that targeting these traits for selection and genetic improvement could be effective in common bean breeding for enhanced Fe nutrition. Additionally, the high Fe bioavailability present in the yellow- and white-seeded genotypes suggests that farmers and consumers need to be informed about the advantage of these seed types in delivering more bioavailable Fe for human health following consumption. The yellow and white genotypes were also the fastest cooking genotypes, adding an additional benefit to the consumer and also promoting appeal (Katuuramu et al., 2020).

The red mottled genotypes Rozi Koko and Chijar had consistently high concentrations of seed Fe but low levels of seed FeBIO. Red mottled common bean genotypes, especially of the Andean gene pool origin, have previously been reported to accumulate more seed Fe than other market classes (Islam et al., 2002; Blair et al., 2010b). These red mottled genotypes may still deliver more absorbable Fe if served with certain foods in the diet since Fe release and absorption is often a function of other nutrients present in the food matrix (Glahn et al., 2017; Diaz-Benito et al., 2018). Previous cell culture, animal, and human investigations have demonstrated that condensed tannins in the seed coats of red-colored dry beans are associated with lower FeBIO in common bean food (Petry et al., 2010; Tako and Glahn, 2011; Wiesinger et al., 2019). Moreover, different classes of seed coat polyphenols have been reported to have promoter (e.g., kaempferol, catechin, kaempferol 3-glucoside, and 3,4-dihydroxybenzoic acid) and inhibitory (e.g., quercetin, myricetin, quercetin 3-glucoside, and myricetin 3-glucoside) effects on iron release and absorption (Hart et al., 2017, 2020). It will be important to comprehensively characterize the different classes of polyphenolic compounds present in the major seed types consumed in Eastern Africa (i.e., red mottled, yellow, and white) as part of common bean biofortification breeding efforts.

Our results revealed that seed Fe was positively correlated with seed Zn concentration. Similar positive associations between seed Fe and Zn have been reported in previous studies in common bean and other crop systems including chickpeas, rice, and sorghum (Blair et al., 2009; Upadhyaya et al., 2012; Phuke et al., 2017; Diaz-Benito et al., 2018). This will be particularly useful in the development of common bean varieties that are high in both seed Fe and Zn concentrations. We actually identified two landrace genotypes, Rozi Koko and Chijar, which had high concentrations of both seed Fe and Zn. This high positive correlation among the two seed minerals suggests that both Fe and Zn might have evolved together and are potentially under similar genetic control of ion transporters and transcription factors related to mineral movement and trafficking in plants and ultimate loading into the seeds (Baxter et al., 2012). Seed FeBIO was not correlated to either seed Fe and Zn concentration as has been previously reported by Katuuramu et al. (2018). Additionally, upon screening of marketplace and breeder collections of common bean from Eastern Africa, it was reported that the Fe concentrations between biofortified and non-biofortified genotypes were similar (Glahn et al., 2020). This suggests that seed Fe concentration is independent of seed FeBIO. Therefore, it will be important in common bean nutrition breeding to target genotypes that result in more deliverable/bioavailable Fe upon consumption. This evidence from our on-farm multilocation trial further strengthens the case that, for nutritious common bean varieties to be developed, the plant breeders will need to actively screen the germplasm and breed for both high seed mineral concentration and FeBIO traits.

## Conclusion

We identified two red mottled genotypes, Rozi Koko and Chijar, with a high combination of both seed Fe and Zn concentrations. Genotypes Blanco Fanesquero and Ervilha had consistently high levels of seed FeBIO. Additionally, the yellow-colored genotype Cebo Cela had both high seed Fe concentration and FeBIO across most of the on-farm field sites. For Uganda’s common bean nutrition breeding, focus should be on the introgression of alleles controlling seed Fe and Zn concentration into the genetic background with high Fe bioavailability. The Manteca bean holds promise to combine high iron concentration with high Fe bioavailability in a market class many consumers in Uganda already prefer because of its fast cooking time.

## Data Availability Statement

The original contributions presented in the study are included in the article/Supplementary Material, further inquiries can be directed to the corresponding author/s.

## Author Contributions

DK contributed to methodology design; collected, analyzed, and visualized the data; and wrote the manuscript. JW collected, analyzed, and visualized the data and contributed to writing and editing of the manuscript. GL and SN organized on-farm trials and contributed to editing of the manuscript. RG contributed to writing and editing of the manuscript. KC conceptualized the study, acquired funding, and contributed to writing and editing of the manuscript. All authors contributed to the article and approved the submitted version.

## Conflict of Interest

The authors declare that the research was conducted in the absence of any commercial or financial relationships that could be construed as a potential conflict of interest.

## Abbreviations

FeBIO: iron bioavailability
LSD: least significant difference
Fe: iron
Zn: zinc
PVE: phenotypic variability explained.
