# Supplementary material for: Investigation of Genotype by Environment Interactions for Seed Zinc and Iron Concentration and Iron Bioavailability in Common Bean
Source: Front Plant Sci. 2021 May 10;12:670965. doi: 10.3389/fpls.2021.670965 (PMC8141707; doi:10.3389/fpls.2021.670965)
Supplement: Supplementary file 1 [file Data_Sheet_1.docx]

Electronic Supplementary Information for Frontiers in Plant Science Journal Article Section Plant Breeding:

**Investigation of Genotype by Environment Interactions for Seed Zinc and Iron Concentration and Iron Bioavailability in Common Bean**

Dennis N. Katuuramu^1,2^, Jason A. Wiesinger^3^, Gabriel B. Luyima^4^, Stanley T. Nkalubo^4^, Raymond P. Glahn^3^, and Karen A. Cichy^1,5*^

^1^ Department of Plant, Soil and Microbial Sciences, Michigan State University, East Lansing, MI, 48824, USA

^2^ USDA - ARS, U.S. Vegetable Laboratory, Charleston, SC, 29414, USA

^3^ USDA - ARS, Robert W. Holley Center for Agriculture and Health, Ithaca, NY, 14853, USA

^4^ Legumes Research Program, National Crops Resources Research Institute, Namulonge, Kampala, Uganda

^5^ USDA - ARS, Sugarbeet and Bean Research Unit, East Lansing, MI, 48824, USA

Supplementary Table S1. Pre-planting soil chemical composition analysis for the nine on-farm study locations over the two years in Uganda.

| District | Location^a^ | Year |  | |  | | Soil nutrient levels at planting | | | | | |  |
| --- | --- | --- | --- | --- | --- | --- | --- | --- | --- | --- | --- | --- | --- |
|  |  |  | pH | OM  (%) | | NO_3_-N  (µg g^-1^) | | Zn  (µg g^-1^) | Fe  (µg g^-1^) | P  (µg g^-1^) | K  (µg g^-1^) | Ca  (µg g^-1^) | Mg  (µg g^-1^) |
| Hoima | KA | 2015 | 6 | 6.7 | | 21.2 | | 1.9 | 7.1 | 12 | 144 | 1,998 | 453 |
|  |  | 2016 | 5.7 | 4.3 | | 5.4 | | 1.4 | 11.0 | 6 | 76 | 1,135 | 223 |
|  | KY | 2015 | 5.9 | 5.6 | | 37.6 | | 1.3 | 8.3 | 8 | 216 | 1,958 | 303 |
|  |  | 2016 | 6.3 | 3 | | 4.4 | | 4.4 | 8.5 | 36 | 45 | 1,482 | 170 |
|  | TU | 2015 | 6.2 | 3.7 | | 22.9 | | 2.1 | 8.2 | 10 | 195 | 1,283 | 183 |
|  |  | 2016 | 5.5 | 6.5 | | 14.9 | | 2.1 | 17.0 | 20 | 212 | 1,107 | 281 |
| Kamuli | KU | 2015 | 6.1 | 3 | | 50.3 | | 2.6 | 9.2 | 12 | 239 | 1,377 | 188 |
|  |  | 2016 | 5.7 | 4.6 | | 16.3 | | 1.9 | 21.0 | 13 | 83 | 1,158 | 237 |
|  | TW | 2015 | 6 | 4.8 | | 8.3 | | 1.0 | 9.0 | 13 | 94 | 1,509 | 278 |
|  |  | 2016 | 5.6 | 2.8 | | 8.9 | | 4.3 | 17.0 | 7 | 68 | 705 | 129 |
|  | GE | 2015 | 6 | 3.6 | | 14.6 | | 3.3 | 12.0 | 20 | 531 | 1,376 | 239 |
|  |  | 2016 | 7.1 | 3.5 | | 11.9 | | 3.1 | 7.8 | 23 | 305 | 2,038 | 222 |
| Rakai | AG | 2015 | 5 | 2.9 | | 25.7 | | 0.9 | 23.0 | 14 | 28 | 444 | 98 |
|  |  | 2016 | 5.5 | 4.3 | | 11.8 | | 2.8 | 22.0 | 23 | 83 | 1,019 | 209 |
|  | KV | 2015 | 5.4 | 4.2 | | 13.5 | | 0.9 | 29.0 | 20 | 63 | 951 | 187 |
|  |  | 2016 | 5.5 | 2.9 | | 6.3 | | 4.2 | 18.0 | 21 | 172 | 833 | 95 |
| Masaka | BA | 2015 | 5.2 | 4.6 | | 22.9 | | 3.0 | 22.0 | 36 | 171 | 1,290 | 298 |
|  |  | 2016 | 5.6 | 5.1 | | 16.4 | | 1.7 | 11.0 | 13 | 124 | 1,379 | 268 |

^a^ Key to location names: KA, Kakindo; KY, Kyamalera; TU, Tugonzagane; KU, Katugezeko; TW, Tweweyo; GE, Tweyunge; AG, Agali-awamu; KV, Kiyovu; and BA, Balitwewunya

Supplementary Table S2. Genotype means for seed zinc concentration (µg g^-1^) of the cooked common bean accessions evaluated at the nine on-farm locations during the 2015 and 2016 field seasons in Uganda.

| Genotype name | Year | Location^a^ | | | | | | | | |
| --- | --- | --- | --- | --- | --- | --- | --- | --- | --- | --- |
|  |  | KA | KY | TU | KU | TW | GE | AG | KV | BA |
| Blanco Fanesquero | 2015 | 28.3 | 27.7 | 27.6 | 30.3 | 22.5 | 30.0 | 23.2 | 24.9 | 27.9 |
|  | 2016 | 27.3 | 30.2 | 35.6 | 32.5 | 20.3 | 27.9 | 31.0 | 24.9 | 26.1 |
| Ervilha | 2015 | 28.0 | 29.6 | 26.3 | 27.7 | 24.4 | 26.4 | 22.5 | 24.4 | 29.0 |
|  | 2016 | 28.2 | 26.8 | 32.6 | 28.9 | 23.6 | 26.5 | 29.7 | 28.7 | 28.4 |
| PI527538 | 2015 | 28.9 | 29.2 | 29.7 | 27.6 | 23.5 | 27.1 | 21.9 | 25.8 | 27.3 |
|  | 2016 | 28.4 | 27.4 | 32.4 | 31.2 | 20.3 | 26.1 | 31.0 | 29.3 | 30.3 |
| Cebo Cela | 2015 | 31.8 | 37.4 | 31.9 | 32.5 | 25.7 | 27.6 | 23.5 | 26.0 | 34.7 |
|  | 2016 | 31.4 | 29.1 | 36.1 | 29.3 | 23.0 | 27.1 | 31.0 | 32.2 | 27.9 |
| Amarelo Cela | 2015 | 27.4 | 28.5 | 26.7 | 28.9 | 27.5 | 29.9 | 24.7 | 26.5 | 27.5 |
|  | 2016 | 25.5 | 25.9 | 29.6 | 29.0 | 19.9 | 24.7 | 32.5 | 30.2 | 27.9 |
| Maalasa | 2015 | 28.4 | 32.1 | 30.3 | 30.9 | 19.1 | 25.0 | 21.3 | 23.9 | 28.4 |
|  | 2016 | 30.3 | 25.6 | 33.4 | 29.9 | 18.0 | 27.6 | 31.9 | 30.3 | 26.3 |
| Rozi Koko | 2015 | 33.6 | 39.6 | 33.3 | 34.4 | 27.6 | 33.6 | 25.2 | 31.4 | 32.2 |
|  | 2016 | 34.9 | 30.6 | 38.9 | 33.5 | 22.9 | 28.1 | 34.5 | 34.8 | 33.4 |
| Chijar | 2015 | 29.6 | 36.4 | 31.8 | 36.2 | 30.1 | 32.2 | 28.8 | 32.9 | 32.3 |
|  | 2016 | 33.5 | 31.6 | 37.0 | 32.9 | 26.5 | 28.8 | 32.4 | 30.9 | 30.8 |
| PR0737-1 | 2015 | 34.6 | 31.9 | 42.0 | 33.2 | 30.2 | 35.4 | 29.3 | 32.5 | 35.9 |
|  | 2016 | 32.2 | 36.2 | 42.0 | 37.5 | 26.2 | 37.3 | 38.1 | 32.1 | 33.6 |
| Vazon 7 | 2015 | 29.2 | 35.2 | 30.7 | 29.6 | 28.2 | 31.2 | 27.2 | 30.5 | 31.5 |
|  | 2016 | 31.1 | 28.4 | 37.3 | 30.7 | 28.6 | 30.6 | 31.9 | 31.1 | 30.1 |
| Kidungu | 2015 | 30.1 | 27.4 | 25.7 | 29.4 | 22.7 | 24.7 | 20.9 | 23.9 | 29.1 |
|  | 2016 | 25.3 | 25.4 | 29.9 | 30.1 | 19.7 | 24.8 | 28.7 | 27.4 | 28.9 |
| Uyole 96 | 2015 | 29.6 | 30.6 | 31.3 | 33.0 | 24.1 | 34.2 | 21.9 | 27.5 | 24.5 |
|  | 2016 | 31.3 | 30.3 | 38.9 | 33.7 | 26.3 | 30.4 | 33.1 | 31.9 | 30.8 |
| Charlevoix | 2015 | 31.8 | 32.8 | 30.5 | 30.6 | 24.6 | 24.5 | 25.0 | 24.7 | 30.8 |
|  | 2016 | 31.9 | 28.2 | 32.2 | 32.6 | 21.2 | 26.2 | 33.7 | 32.8 | 29.6 |
| Selian 97 | 2015 | 30.2 | 33.1 | 27.1 | 29.9 | 26.4 | 29.3 | 26.3 | 27.2 | 32.0 |
|  | 2016 | 28.0 | 28.5 | 33.6 | 29.3 | 23.4 | 25.3 | 37.4 | 31.4 | 30.9 |
| Sacramento | 2015 | 36.3 | 33.8 | 30.9 | 28.4 | 26.0 | 27.0 | 23.3 | 25.0 | 30.7 |
|  | 2016 | 29.3 | 29.1 | 31.4 | 31.3 | 22.2 | 27.5 | 30.6 | 30.0 | 28.1 |
| *Local checks:* |  |  |  |  |  |  |  |  |  |  |
| NABE-15 | 2015 | 26.9 | 30.7 | 29.4 | - | - | 24.1 | - | 23.5 | 29.2 |
|  | 2016 | 29.0 | 27.0 | 34.8 | - | - | 28.3 | - | 35.5 | 32.0 |
| NABE-4 | 2015 | - | - | - | - | - | - | 27.3 | - | - |
|  | 2016 | - | - | - | - | - | - | 38.5 | - | - |
| Masindi yellow | 2015 | - | - | - | 28.0 | 23.3 | - | - | - | - |
|  | 2016 | - | - | - | 29.0 | 21.6 | - | - | - | - |

^a^ Key to location names: KA, Kakindo; KY, Kyamalera; TU, Tugonzagane; KU, Katugezeko; TW, Tweweyo; GE, Tweyunge; AG, Agali-awamu; KV, Kiyovu; and BA, Balitwewunya

Supplementary Table S3. Genotype means for seed iron concentration (µg g^-1^) of the cooked common bean accessions evaluated at the nine on-farm locations during the 2015 and 2016 field seasons in Uganda.

| Genotype name | Year | Location^a^ | | | | | | | | |
| --- | --- | --- | --- | --- | --- | --- | --- | --- | --- | --- |
|  |  | KA | KY | TU | KU | TW | GE | AG | KV | BA |
| Blanco Fanesquero | 2015 | 65.7 | 59.5 | 60.1 | 76.9 | 61.0 | 73.3 | 49.7 | 61.8 | 62.3 |
|  | 2016 | 65.9 | 71.5 | 76.5 | 74.7 | 55.4 | 62.2 | 78.9 | 78.6 | 65.4 |
| Ervilha | 2015 | 62.0 | 61.7 | 49.2 | 61.4 | 54.4 | 63.9 | 48.0 | 63.0 | 58.1 |
|  | 2016 | 62.4 | 66.4 | 70.8 | 62.6 | 54.6 | 62.5 | 67.6 | 73.3 | 63.1 |
| PI527538 | 2015 | 53.9 | 55.5 | 56.5 | 70.8 | 52.5 | 65.2 | 48.0 | 65.9 | 50.6 |
|  | 2016 | 64.8 | 67.1 | 73.8 | 67.2 | 49.5 | 59.5 | 71.1 | 80.4 | 67.9 |
| Cebo Cela | 2015 | 70.6 | 83.6 | 64.7 | 82.4 | 78.9 | 73.3 | 61.0 | 71.1 | 79.7 |
|  | 2016 | 70.3 | 82.1 | 88.6 | 75.6 | 61.1 | 69.2 | 65.8 | 85.5 | 63.4 |
| Amarelo Cela | 2015 | 55.3 | 67.2 | 55.2 | 60.5 | 57.1 | 66.5 | 64.3 | 71.4 | 60.5 |
|  | 2016 | 65.1 | 66.3 | 64.6 | 63.7 | 53.3 | 68.0 | 57.1 | 78.0 | 69.4 |
| Maalasa | 2015 | 67.0 | 62.1 | 54.9 | 66.6 | 53.1 | 71.9 | 40.7 | 61.9 | 54.8 |
|  | 2016 | 70.2 | 67.5 | 76.1 | 63.3 | 57.8 | 64.9 | 59.8 | 79.2 | 63.0 |
| Rozi Koko | 2015 | 69.5 | 78.2 | 65.2 | 81.9 | 73.8 | 85.7 | 53.7 | 75.0 | 66.7 |
|  | 2016 | 78.6 | 77.9 | 86.2 | 79.5 | 59.4 | 70.6 | 79.3 | 96.7 | 79.0 |
| Chijar | 2015 | 62.8 | 75.4 | 63.1 | 78.1 | 64.5 | 74.3 | 68.6 | 73.7 | 68.7 |
|  | 2016 | 74.1 | 73.3 | 81.1 | 71.5 | 66.4 | 92.9 | 71.0 | 77.7 | 72.5 |
| PR0737-1 | 2015 | 53.3 | 56.5 | 77.9 | 71.7 | 64.0 | 68.6 | 53.7 | 74.6 | 65.1 |
|  | 2016 | 65.5 | 73.6 | 77.9 | 70.8 | 59.8 | 78.1 | 68.5 | 68.5 | 65.8 |
| Vazon 7 | 2015 | 49.8 | 53.7 | 50.7 | 63.9 | 56.0 | 61.5 | 58.0 | 64.4 | 56.0 |
|  | 2016 | 61.4 | 59.1 | 62.0 | 54.6 | 61.6 | 64.6 | 55.9 | 66.8 | 62.8 |
| Kidungu | 2015 | 60.1 | 49.1 | 44.7 | 61.8 | 56.2 | 56.7 | 47.0 | 56.6 | 59.9 |
|  | 2016 | 59.0 | 55.7 | 63.6 | 63.5 | 49.6 | 58.4 | 54.9 | 63.6 | 57.8 |
| Uyole 96 | 2015 | 62.2 | 61.2 | 63.8 | 83.3 | 64.5 | 91.6 | 44.1 | 67.3 | 56.2 |
|  | 2016 | 71.1 | 72.1 | 86.8 | 70.7 | 63.0 | 65.3 | 72.5 | 83.5 | 73.7 |
| Charlevoix | 2015 | 63.9 | 55.6 | 57.3 | 64.3 | 51.9 | 57.6 | 50.5 | 63.4 | 64.9 |
|  | 2016 | 71.5 | 65.7 | 68.1 | 69.5 | 67.4 | 59.9 | 70.8 | 88.1 | 67.9 |
| Selian 97 | 2015 | 57.6 | 61.1 | 52.8 | 62.3 | 60.3 | 66.7 | 51.1 | 64.5 | 62.3 |
|  | 2016 | 63.8 | 76.1 | 77.8 | 73.3 | 61.1 | 65.5 | 70.1 | 80.1 | 70.4 |
| Sacramento | 2015 | 64.9 | 59.1 | 50.5 | 57.7 | 56.1 | 60.6 | 48.6 | 60.3 | 59.8 |
|  | 2016 | 60.5 | 63.8 | 67.3 | 70.1 | 61.2 | 59.2 | 54.5 | 75.8 | 61.0 |
| *Local checks:* |  |  |  |  |  |  |  |  |  |  |
| NABE-15 | 2015 | 50.8 | 59.6 | 51.1 | - | - | 53.8 | - | 57.9 | 58.4 |
|  | 2016 | 64.5 | 59.6 | 74.7 | - | - | 61.7 | - | 87.0 | 75.0 |
| NABE-4 | 2015 | - | - | - | - | - | - | 66.5 | - | - |
|  | 2016 | - | - | - | - | - | - | 63.8 | - | - |
| Masindi yellow | 2015 | - | - | - | 57.1 | 46.6 | - | - | - | - |
|  | 2016 | - | - | - | 57.5 | 50.4 | - | - | - | - |

^a^ Key to location names: KA, Kakindo; KY, Kyamalera; TU, Tugonzagane; KU, Katugezeko; TW, Tweweyo; GE, Tweyunge; AG, Agali-awamu; KV, Kiyovu; and BA, Balitwewunya

Supplementary Table S4. Genotype means for seed iron bioavailability (% of Merlin navy bean control) of the cooked common bean accessions evaluated at the nine on-farm locations during the 2015 and 2016 field seasons in Uganda.

| Genotype name | Year | Location^a^ | | | | | | | | |
| --- | --- | --- | --- | --- | --- | --- | --- | --- | --- | --- |
|  |  | KA | KY | TU | KU | TW | GE | AG | KV | BA |
| Blanco Fanesquero | 2015 | 85.5 | 96.5 | 96.5 | 78.0 | 72.0 | 83.0 | 69.5 | 92.5 | 76.0 |
|  | 2016 | 71.0 | 83.0 | 116.0 | 67.5 | 83.0 | 75.0 | 66.0 | 94.5 | 64.0 |
| Ervilha | 2015 | 84.0 | 93.5 | 97.5 | 77.5 | 106.5 | 92.5 | 76.0 | 102.5 | 75.0 |
|  | 2016 | 83.5 | 87.0 | 109.5 | 98.0 | 78.0 | 101.0 | 81.5 | 83.5 | 75.5 |
| PI527538 | 2015 | 34.0 | 61.0 | 45.5 | 36.5 | 25.0 | 43.5 | 28.0 | 8.5 | 34.5 |
|  | 2016 | 57.5 | 18.5 | 53.0 | 23.5 | 35.5 | 41.0 | 56.0 | 27.0 | 45.0 |
| Cebo Cela | 2015 | 84.0 | 112.0 | 86.0 | 76.0 | 89.5 | 81.0 | 70.5 | 92.0 | 85.5 |
|  | 2016 | 74.0 | 90.5 | 72.0 | 97.5 | 77.0 | 84.0 | 76.0 | 88.5 | 71.5 |
| Amarelo Cela | 2015 | 29.0 | 51.5 | 43.5 | 15.0 | 27.5 | 40.5 | 23.0 | 8.5 | 29.0 |
|  | 2016 | 52.5 | 14.5 | 44.0 | 20.5 | 34.5 | 39.0 | 51.5 | 31.5 | 45.0 |
| Maalasa | 2015 | 39.5 | 63.5 | 47.5 | 29.0 | 28.5 | 58.5 | 23.0 | 14.5 | 29.5 |
|  | 2016 | 55.5 | 28.0 | 55.5 | 41.5 | 42.0 | 58.0 | 54.0 | 35.5 | 39.5 |
| Rozi Koko | 2015 | 46.0 | 62.5 | 49.0 | 38.5 | 31.0 | 52.0 | 26.0 | 34.0 | 41.5 |
|  | 2016 | 52.5 | 44.5 | 41.0 | 47.0 | 40.5 | 55.0 | 56.0 | 43.5 | 59.5 |
| Chijar | 2015 | 37.5 | 62.0 | 48.5 | 19.5 | 16.5 | 36.0 | 23.0 | 8.5 | 33.5 |
|  | 2016 | 50.0 | 14.5 | 55.0 | 23.0 | 31.5 | 32.0 | 51.0 | 29.5 | 48.0 |
| PR0737-1 | 2015 | 31.5 | 61.5 | 55.0 | 26.0 | 23.0 | 40.0 | 19.0 | 8.0 | 38.5 |
|  | 2016 | 49.5 | 18.5 | 55.0 | 32.0 | 38.0 | 41.0 | 58.5 | 29.5 | 52.5 |
| *Local checks:* |  |  |  |  |  |  |  |  |  |  |
| NABE-15 | 2015 | 43.5 | 85.5 | 56.0 | - | - | 58.0 | - | 14.0 | 43.0 |
|  | 2016 | 60.0 | 29.5 | 60.5 | - | - | 46.5 | - | 43.5 | 54.5 |
| NABE-4 | 2015 | - | - | - | - | - | - | 30.0 | - | - |
|  | 2016 | - | - | - | - | - | - | 57.0 | - | - |
| Masindi yellow | 2015 | - | - | - | 42.5 | 27.5 | - | - | - | - |
|  | 2016 | - | - | - | 34.5 | 43.0 | - | - | - | - |

^a^ Key to location names: KA, Kakindo; KY, Kyamalera; TU, Tugonzagane; KU, Katugezeko; TW, Tweweyo; GE, Tweyunge; AG, Agali-awamu; KV, Kiyovu; and BA, Balitwewunya
